# Co-Occurrence of Taste and Odor Compounds and Cyanotoxins in Cyanobacterial Blooms: Emerging Risks to Human Health?

**DOI:** 10.3390/microorganisms11040872

**Published:** 2023-03-28

**Authors:** Maura Manganelli, Emanuela Testai, Zakaria Tazart, Simona Scardala, Geoffrey A. Codd

**Affiliations:** 1 Istituto Superiore di Sanità, Department of Environment and Health, viale Regina Elena, 299, 00162 Rome, Italy; emanuela.testai@iss.it (E.T.); simona.scardala@iss.it (S.S.); 2 Department of Food Sciences and Nutrition, University of Malta, 2080 Msida, Malta; zakaria.tazart@gmail.com; 3 School of Natural Sciences, University of Stirling, Stirling FK9 4LA, UK; g.a.codd@stir.ac.uk; 4 School of Life Sciences, University of Dundee, Dundee DD1 5EH, UK

**Keywords:** cyanobacteria, geosmin, 2-methylisoborneol, β-ionone, β-cyclocitral, microcystins, cylindrospermopsins, neurotoxins, risk assessment, water treatment

## Abstract

Cyanobacteria commonly form large blooms in waterbodies; they can produce cyanotoxins, with toxic effects on humans and animals, and volatile compounds, causing bad tastes and odors (T&O) at naturally occurring low concentrations. Notwithstanding the large amount of literature on either cyanotoxins or T&O, no review has focused on them at the same time. The present review critically evaluates the recent literature on cyanotoxins and T&O compounds (geosmin, 2-methylisoborneol, β-ionone and β-cyclocitral) to identify research gaps on harmful exposure of humans and animals to both metabolite classes. T&O and cyanotoxins production can be due to the same or common to different cyanobacterial species/strains, with the additional possibility of T&O production by non-cyanobacterial species. The few environmental studies on the co-occurrence of these two groups of metabolites are not sufficient to understand if and how they can co-vary, or influence each other, perhaps stimulating cyanotoxin production. Therefore, T&Os cannot reliably serve as early warning surrogates for cyanotoxins. The scarce data on T&O toxicity seem to indicate a low health risk (but the inhalation of β-cyclocitral deserves more study). However, no data are available on the effects of combined exposure to mixtures of cyanotoxins and T&O compounds and to combinations of T&O compounds; therefore, whether the co-occurrence of cyanotoxins and T&O compounds is a health issue remains an open question.

## 1. Introduction

Cyanobacteria are oxygenic phototrophic, Gram-negative prokaryotes, that can produce large blooms in surface water bodies. These blooms are increasing in magnitude, geographical spread, frequency and duration due to climate change and eutrophication [1]. Cyanobacteria are very well known for the production of a vast array of secondary bioactive metabolites, some of which may have the potential for useful applications in the pharmaceutical, food, cosmetic, agriculture and energy sectors, while others have toxic effects on humans and animals (cyanotoxins: CTX) (see, for example, these two extensive databases and references therein: [2,3]). Toxic cyanobacterial blooms, and related cyanobacterial mass accumulations (scums and mats), in reservoirs or waterbodies used as drinking water sources for humans and domestic and wild animals or for fisheries and recreational activities, can present hazards to human and animal health and also problems for the environment from a One Health perspective [4,5]. Concerns related to exposure to the most commonly occurring and best characterized CTX have been described and extensively reviewed by the World Health Organization (WHO), which proposed for some of them, for which the toxicological database is sufficiently robust, specific guideline values for potential human exposure via drinking water and recreational activities [6,7,8,9].

Among the produced metabolites, some are volatile compounds causing bad tastes and odors (T&O) in inadequately treated drinking water at very low concentrations, e.g., the threshold of perception in humans is around 10 ng/L for terpenoids, which are included among T&O compounds. The latter are generally considered to have no toxic effects for humans via oral exposure when present in drinking water since the thresholds for inducing adverse effects are well above their odor threshold concentrations, which makes drinking this water unacceptable for consumption due to organoleptic characteristics, thus avoiding human exposure [10,11]. However, they can cause important economic problems for drinking water providers where they aim to supply tasteless and odorless drinking water and also to aquaculture plants. Finfish and shellfish harvested from freshwater and estuarine/marine facilities may indeed acquire a bad taste and smell, becoming inedible. The removal of off-flavors in farmed fish can cause additional annual costs of 10–60 million US dollars for producers [12]. By causing unpleasant odors, even in recreational water bodies, T&O compounds could also represent a relevant socioeconomic problem causing significant loss to the tourism industry.

Problems with CTX and T&O for the supply of tap drinking water are related to the low efficiency of their removal by standard drinking water treatment processes: they can remove chlorophyll-a with an efficiency of 100%, but T&O compounds and microcystins (one of the most commonly encountered classes of CTX) cannot be removed with the same efficiency. Shang et al. [13] and Zamyadi et al. [14] determined thresholds for chlorophyll-a or cyanobacterial cell abundance to keep CTX and T&O below WHO guideline values or odor threshold concentrations. These thresholds are different based on dominant species [13] and the kind of pre- and water treatment plants [14] since many of the utilized treatments had a very low efficiency.

Humans can be exposed to CTX via drinking water, accidental ingestion of water during recreational activities, via contaminated food and food supplements, via dermal contact and potentially via inhalation of aquatic aerosols and dust [15,16,17]. The risk of exposure to CTX is presently managed by considering various exposure scenarios [5]. On the contrary, given that no or very limited toxic effects have been observed in aquatic organisms or in in vitro testing at environmentally relevant concentrations of T&O compounds and that people avoid the use of bad-smelling and tasting water, the presence of T&O substances has been considered an issue only for drinking water treatment plant management (since most of them resort to conventional treatment), aquaculture plants, or for the economic loss due to foul-smelling recreational environments, but not as an actual human health risk. However, people could be exposed to many different T&O substances, as with CTX, during recreational activities, through accidental ingestion of water or by eating contaminated food if taste or odor are masked by the presence of a mixture that changes the threshold of perception or the fragrance of the compound [10]. 

Recently, data have been published on possible adverse effects upon toxicity test organisms of three of the most important and diffuse T&O compounds produced by cyanobacteria: geosmin (GEO), 2-methylisoborneol (MIB) and β-ionone, at environmentally relevant concentrations [18,19,20], leading to an emerging concern for human health, especially for susceptible population subgroups including children, who are not repelled by playing and swimming in dirty water, as well as for animal pets and/or livestock and wild animals. Since a number of different T&O chemicals, some not yet characterized, can be produced at the same time and concurrently with CTX, another relevant emerging issue to be considered should be co-exposure to a mixture of CTX and T&O compounds during the same activities.

Various reviews have listed tens of T&O compounds that can be naturally produced by cyanobacteria, algae, some actinobacteria and myxobacteria (e.g., [21,22,23]); many of the reviews are focused on early detection methods for the proactive management of drinking water treatment plants [24] or on the more efficient multibarrier treatments to remove cyanobacterial cells and their metabolites, including T&O compounds from source water within drinking water plants [14]. Several more reviews have been published on the CTXs (dealing with genetics, toxicological profiles and biochemical pathways) (e.g., [5,15,16,25,26]). However, most of the studies or reviews have focused separately on CTX or T&O. Indeed, despite some field studies having demonstrated the co-occurrence of CTX and T&O compounds, only a few lab studies, with either field samples or axenic monocyanobacterial strains have investigated or confirmed the co-production of T&O compounds and CTX by the same cyanobacterial strain. Thus, the co-occurrence of the two groups of chemicals has only very rarely been addressed simultaneously, and neither have such combinations been analyzed for their potential combined effects when co-occurring. The fact that only a few field studies have focused on the production of both CTX, and T&O is probably due to technical difficulties in tracking the T&O compound sources unambiguously since various potential T&O-producing species often co-occur in the same community and within cyanobacterial biomass: bloom, scum and mat-developments. These problems could be partially overcome by applying a multiphasic approach, which combines morphological identification with molecular techniques aimed at recognizing species and substance-producing genes [21], which, in the authors’ opinion, should be paralleled with the specific and quantitative analysis of CTX and T&O compounds. One of the difficulties for drinking water providers, and managers of recreational waters and aquaculture facilities is the lack of predictive models able to forecast toxic cyanobacterial blooms. The possibility of using T&O compounds as an early warning has been proposed to implement preventative action to avoid increased risk exposure and the inadequate or malfunctioning of the treatment plant; however, this possibility has never been confirmed as valid so far.

Based on the emerging relevance of considering these two groups of compounds together, this paper aims to critically review the recent literature on CTX and T&O compounds and their possible interactions to assess the state of the combinatorial concept and to identify research needs to protect human and animal health from risky exposure to cyanobacterial metabolites. Studies on the co-occurrence of T&O and CTX in environmental cyanobacterial mass populations, including blooms, and using defined laboratory cultures are assessed to see, for example, if the production of CTX and T&O compounds is due to the same cyanobacterium or to two different strains or to two different organisms and to identify factors which influence them. The ultimate objectives are to verify whether T&O compounds can be used as warning signals for the development and presence of toxic/non-toxic blooms and if mixtures of T&O and CTX can present potential toxicity other than that of the single chemicals alone.

## 2. Cyanotoxins (CTXs) and Taste and Odor (T&O) Compounds: Characteristics and Toxicity

### 2.1. CTXs

The most widely found and best characterized CTX, their cyanobacterial sources, and a range of their intracellular concentrations are listed in Table 1. For detailed information on their toxicological profiles, occurrence and producing organisms, see Chorus and Welker [5], Buratti et al. [16] and Bernard et al. [27]. A revision of the available toxicological information, analysis of exposure scenarios, and updated provisional guidance values for short- and long-term exposures has been provided for drinking water as well as for exposures to different CTX as updated by Chorus and Welker [5] and summarized in Table 2. It is notable that the derived Guidance Values were considered provisional for the cyanotoxins due to database deficiency. GVs can be applied to total MCs, total CYNs and total STXs due to the lack of data for different variants. The characteristics of the different CTX groups are only briefly reviewed here.

#### 2.1.1. Microcystins and Nodularins

**Figure 1:**
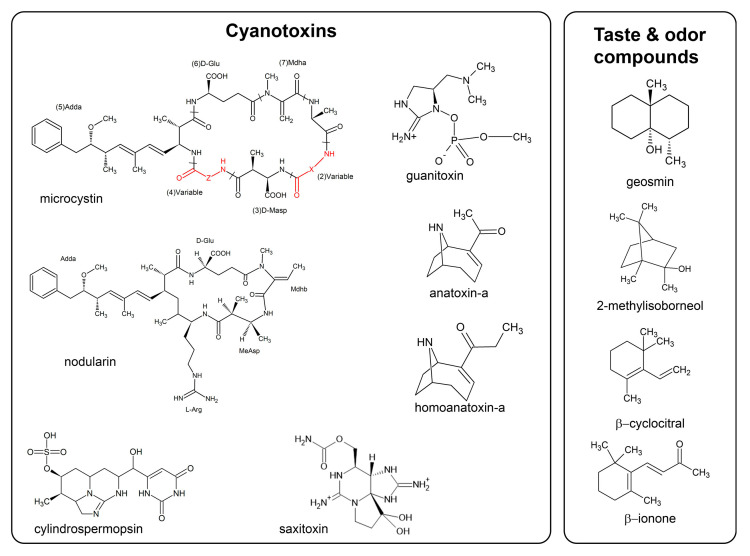
Structures of the cyanotoxins and taste and odor compounds considered in the review. The red part in the microcystin structure shows the positions 2 and 4 of the ring, whose variable amino acids characterize different commonly occurring variants. Further aminoacid substitutions and modifications throughout the microcystins ring generate additional variants.

Microcystins (MCs) and nodularins (NODs) (Figure 1) are two classes of cyclic hepta- and penta peptides, respectively and are primarily known for their hepatotoxicity. MCs are the most commonly reported CTX worldwide, and >310 variants are known [3]. Their structure is characterized by the presence of the amino acid Adda [(2S,3S,8S,9S)-3-amino-9-methoxy-2,6,8-trimethyl-10-phenyl-4,6-decadienoic acid], and the variants are characterized by substitution of amino acids mainly at positions 2 and 4 of the ring and by other modifications including demethylation in various positions. MCs are not able to passively cross the eukaryotic cell membrane but enter the cell via transport proteins. Intestinal absorption is the result of the net balance between the activity of influx proteins, which include, but are not limited to the organic anion transporters, OATP1A2 and 2B1, as mainly expressed in the gut versus the action of efflux proteins such as MRP2 and BCRP, but not Pgp [28,29]. Since the liver has a high expression of OATPs, mainly OATP1B1 and 1B3, MCs accumulate in that organ, accounting for their primary hepatotoxicity. However, additional toxicity has been reported in those tissues expressing OATPs, although the critical effect considered for the derivation of guideline values for health protection versus MCs is their hepatotoxicity. Studies showing potential toxicity for organs other than the liver have been considered not robust enough to provide a new Point of Departure (PoD) for any health-based reference value due to some weaknesses in experimental design and reporting [8,17]. The molecular initiating event for the toxicity of MCs is the inhibition of the eukaryotic serine/threonine phosphatases (PP1 and, to a larger extent, PP2A) [30]. This inhibition causes phosphorylation/dephosphorylation imbalance of key control proteins that regulate multiple signaling pathways, thus triggering a cascade of effects (e.g., cytoskeleton alterations, lipid peroxidation, oxidative stress, mitochondrial disruption and apoptosis), which can culminate in cell death or proliferation depending on dose [16]. Indeed, acute exposure to high doses of MCs causes hemorrhage in the liver, while after prolonged exposure to low doses (below 20 μg/kg bw [31]) MCs may exert other effects, including tumor promotion [5,15,16,32]. Acute toxic potencies vary among MC variants [33,34]. These differences are attributed to the route- and variant-specific toxicokinetics [35,36] since many studies have reported similar toxicodynamics, including PP-inhibition potency for MC variants [8].

Only 10 variants of nodularin are known. While MCs are produced by many different cyanobacterial genera and species (Table 1) and are the most widely found CTXs, NODs are produced mainly by species of the genus *Nodularia*, principally *Nodularia spumigena*, which is generally limited to coastal-brackish environments. NOD-producing aquatic and terrestrial species of *Nostoc* also occur [16,27]. On the basis of their structural similarity with MCs, as well as the same initial step of their mode of action (i.e., protein phosphatase inhibition), NODs are often considered together with MCs, and their guideline values for drinking water are often considered the same (e.g., [37]). However, some differences appear to exist, starting with the non-covalent nature of the binding with PP2A [38]. NODs have been reported to be tumor promoters without the requirement for an exogenous initiator (see [5]), but the scant amount of data available led the International Agency for Research on Cancer (IARC) to indicate that NODs are classifiable as Group 3 (meaning they are not classifiable as to their carcinogenicity to humans) [39], while MC-LR is classified as a class 2B carcinogen (that is, possibly carcinogenic to humans due to the demonstrated tumor promoting activity) [39].

MCs and NODS are generally water soluble, although their logP can vary [36]. They are extremely stable even after boiling [40]. Indeed, the commonly occurring, high-toxicity variant MC-LR remains stable for up to 4 days at 100 °C and for at least 15 days at 20 °C, from pH1 to pH10 [41].

#### 2.1.2. Cylindrospermopsins

The cylindrospermopsin (CYN) (Figure 1) molecule consists of a tricyclic guanidine group combined with a hydroxymethyl uracil; the known family comprises five variants, with CYN as the most widely identified. It is considered a cytotoxin since it produces effects in different mammalian organs, although hepato- and nephrotoxicity are the ‘critical’ effects considered to derive the provisional TDI by WHO [7]. The mechanisms involved in CYN toxicity in different organs are not fully elucidated, although protein synthesis inhibition has been evidenced in in vivo and in vitro studies attributed to the parent toxin, at least in the liver [7,16]. Since the use of cytochrome P450 inhibitors decreased CYN toxicity [42] without decreasing protein synthesis inhibition [43], it has been hypothesized that the parent toxin and the possibly formed metabolites of CYN could exert toxicity by different mechanisms, depending on CYN concentrations. However, these metabolites considered by some authors as potentially genotoxic have not yet been identified.

CYNs are produced by strains of various cyanobacterial genera and species [27], primarily in the order Nostocales (Table 1). Known CYN producers within the order of Oscillatoriales include *Microseira* (formerly *Lyngbya*) and *Oscillatoria*, many of which are primarily benthic [27]. The distribution of CYN-producing strains of some species follows a geographic pattern, which may have changed in recent decades: whereas *Raphidiopsis raciborskii* is a major CYN-producer in Australia, New Zealand and Asia, strains of this species from Europe, the Americas and Africa do not synthesize CYNs. In Europe, CYN production appears to be largely confined to *Aphanizomenon* spp. and *Dolichospermum* spp. [7].

#### 2.1.3. Anatoxins and Saxitoxins

Anatoxins and saxitoxins (Figure 1) are neurotoxic alkaloids. Although acting via different mechanisms, all of the known cyanobacterial neurotoxins act on the neuromuscular system by blocking skeletal and respiratory muscles, leading to death by respiratory failure [15,26,44].

Anatoxin-a (ATX) and its analog homoanatoxin-a (HomoATX) bind to nicotinic receptors for acetylcholine in the central and peripheral nervous system and in neuromuscular junctions with a pre- and post-synaptic depolarizing action. This triggers neurotransmitter release with increased stimulation of postsynaptic receptors, enhanced by ATX resistance to enzymatic hydrolysis (by acetylcholinesterase), and death is due to muscular paralysis and respiratory failure [26,45]. Limited evidence suggests that the analog HomoATX acts in the same way with a similar potency [6]. ATXs have often been linked to the deaths of dogs and wild animals after drinking contaminated water and/or grooming themselves after swimming in contaminated water. Dogs may also be attracted to ingest fragments of wet and dry shoreline mats of ATX-containing benthic cyanobacteria [46]. ATXs are produced by strains of various genera and species of cyanobacteria found primarily in freshwater environments (Table 1) [27], many of which are benthic. These neurotoxins are produced in many geographical areas by a variety of taxa belonging to Nostocales and to Oscillatoriales. Interestingly, the American and European isolates of *Dolichospermum circinale* investigated so far produce only ATX, while the Australian isolates of this species exclusively produce saxitoxins, even if the corresponding strains are reported to form a phylogenetically coherent group [47].

Saxitoxins (STX), also known as paralytic shellfish poisoning toxins (PSP), can be produced by cyanobacteria and also by some marine dinoflagellates. STX is a family of at least 57 analogs, which have all, to date, been found to be hydrophilic except one, produced by the freshwater cyanobacterium *Lyngbya wollei*. They block Na^+^ channels in neuronal cells and Ca^++^ and K^+^ channels in cardiac cells: the typical neurological symptoms are nervousness, twitching, ataxia, convulsions and muscle and respiratory paralysis, leading at a lethal dose to death in a variable time of 2–24 h [48]. Since around 50% of the natural STX variants within the group share the same mechanism, it has been suggested that it could be the same for all of this group. However, metabolic interconversions between variants have been reported [26]; for example, sulfated STXs present in water can give rise to the more toxic STX and neoSTX in seafood tissues, resulting in more severe poisoning episodes than expected if only the toxins present in the water are considered. The production of STX congeners is strain-specific and depends on the geographical area of production. The cyanobacterial producers are in the families of Nostocales and Oscillatoriales.

Another neurotoxin considered by WHO as a potential contaminant in drinking and recreational water is guanitoxin (GNTX) [15,49]. GNTX, was formerly known as anatoxin-a(S) since it was isolated from a strain of *Anabaena*, the “S” denoting the hypersalivation caused by this toxin in mammals [49]. GNTX is structurally totally different from the alkaloid ATX, being an organophosphorus compound. At present, no structural analogs of GNTX are known. As a naturally occurring organophosphate, GNTX irreversibly inhibits acetylcholinesterase in neuro-muscular junctions, blocking the hydrolysis of the neurotransmitter, leading to nerve hyperexcitability and to death by respiratory arrest [26]. Data on oral administration as well as on subchronic and/or chronic toxicity, are not available. GNTX is produced by a few species of *Dolichospermum* (formerly named *Anabaena*). Relatively limited attention has been given to the environmental occurrence of this toxin. The lack of analytical standards and methods, GNTX instability and fewer validated poisoning events may all have combined to restrict adequate awareness of the environmental occurrence and significance of this neurotoxin.

#### 2.1.4. Other Bioactive Peptides and CTX Biosynthesis

In addition to the most studied CTXs described above, cyanobacteria produce an incredible array of other bioactive peptides, and some classes are beginning to raise a concern about their potential toxicity, including cyanopeptolins, aeruginosins, anabaenopeptins and microginins. These molecules occur at concentrations similar to those of MCs and inhibit many peptidases at nanomolar concentrations. Most of these known metabolites, including the known CTXs, are synthesized by three biosynthetic pathways or hybrids thereof: nonribosomal peptide synthetases (NRPS), polyketide synthases (PKS) or via the ribosomal synthesis of peptides that are modified post-translationally [50,51]. A broad chemical diversity is facilitated by these complex pathways at relatively metabolic little expense, by variations of amino acids in a few positions, modifications such as methylation or dehydration, and others, upon a few basic structures [52,53]. The lack of available standards and appropriate analytical techniques is constraining the study of these compounds [54]. Such plasticity maintains a high genetic and chemical polymorphism, as has been observed in field populations of *Microcystis* and *Planktothrix* [55,56]. This plasticity of bioactive product formation may be a requirement to respond to an environmental “arms race” between species-specific predators and parasites [53,55,57], as well as providing strategies to withstand stressful environmental conditions [25]. Indeed, also for the known CTXs, it has been shown that in the same population/community, there is a variable share of potentially toxic (i.e., carrying gene clusters for CTX biosynthesis) versus non-toxic strains (without the gene or carrying a deletion which inactivates the genes) which may have a different fitness with respect to changing environmental parameters (pCO_2_, N concentration/chemical speciation, light/shade during the bloom, etc.) [25]. Finally, cyanobacteria can rapidly modulate the expression of CTX genes [58].

### 2.2. T&O Compounds

Taste and odor molecules (T&O) are mostly volatile organic compounds (VOCs), and an extensive list of these compounds has been attributed to different biological sources and environments [21,59,60]. The WHO drinking water guidelines [11] report a list of such compounds of both anthropogenic (e.g., xylenes and toluene) and natural origin (e.g., geosmin) that can change the taste, odor or appearance of drinking water at concentrations well below those which can cause adverse health effects, therefore for most of them, no Guideline Values have been derived. In addition to analytical difficulties in determining which compounds give rise to T&O events because of low odor and taste thresholds, another confounding element may be the fact that mixtures of T&O compounds result in odors or tastes that differ from individual substances, producing a synergistic effect on the odor produced [61]. However, only a few molecules produced by cyanobacteria are frequent causes of important T&O events in water supplies, and this review focuses on -them.

#### 2.2.1. Geosmin (GEO) and 2-Methylisoborneol (MIB)

Among the naturally produced T&O compounds, the terpenoids geosmin (GEO) (2β,6α-dimethylbicyclo [4.4.0]decan-1β-ol) and 2-methylisoborneol (MIB) (Figure 1) have been associated with the global majority of waterborne T&O episodes [62] and have been most studied. They are the only T&O compounds included in the revised (2nd) edition of the WHO derivations and recommendations for the health management of cyanobacteria and CTX in water [5]. Since the T&O toxicological thresholds are well above their odor threshold concentration (OTC) [63] and people not drinking water with an unpleasant odor or taste are not orally exposed to the compounds, WHO considers that no guidance value needs to be defined [64].

GEO and MIB present a characteristic earthy/musty odor, with an additional moldy odor due to MIB, that can be transferred to water and to edible fish raised in aquaculture. Due to their lipophilic properties, both MIB and GEO easily cross the gills and guts of fish and can be absorbed from the fish intestinal tract after ingestion of the T&O-producer organisms, causing long depuration times and higher expense for the removal of the bad flavors accumulated in the flesh before commercialization [65]. Each compound exists as (+) and (−) enantiomers, but biological sources produce the (−) stereoisomer, which is 10 times more potent than its (+) counterpart [66]. The commercially available standards are a racemic mixture of the two isomers and have odor threshold concentrations (OTC) around 10 ng/L, which could be an overestimation, given the lower activity of the (+) stereoisomer [67]. MIB and GEO are produced not only by many cyanobacteria but also by some fungi, actinobacteria and myxobacteria ([22] and references therein). Tracking their origin during waterborne T&O events has been very difficult because of the co-occurrence of the many potential T&O-producing microorganisms. However, several specific molecular probes are now available to identify and discriminate between cyanobacteria and other organisms [68,69,70,71,72,73,74].

Both GEO and MIB are produced by cyanobacteria as secondary metabolites during active growth and are generally released only after cell lysis and decay. They are synthetized by the 2-C-methyl-D-erythritol 4-phosphate (MEP) pathway of isoprenoid production (e.g., steroids, hormones and photosynthetic pigments including chlorophyll *a* and carotenoids), although there are key differences in the enzymes used in the final steps of their biosynthesis. Only one geosmin synthase (encoded by the *geoA* gene), a bi-functional terpene synthase, is needed to catalyze the formation of GEO from farnesyl diphosphate (FPP), while two enzymes, expressed by a two-gene operon are necessary to convert geranyl diphosphate (GPP) into MIB [22]: first, a C-methyl transferase to catalyze the methylation of GPP into 2-methyl-GPP and secondly a monoterpene synthase to catalyze the cyclization of 2-methyl-GPP into MIB.

Several MIB operons and *geoA* genes have been identified from various streptomyces, actinomycetes, myxobacteria and cyanobacteria. Churro et al. [75] have shown, by a detailed phylogenetic analysis, that the *geoA* gene is scattered throughout a few species of three bacterial phyla: the Cyanobacteria, Actinomycetes and Proteobacteria (delta and gamma classes). These authors suggest that the *geoA* gene would have been of very ancient, diffuse origin among the bacteria and that it was later lost by negative pressure selection. As for genes encoding for CTX biosynthesis, the presence of the *geoA* gene and its expression is strain specific and can be regulated by environmental factors (see below).

Among the cyanobacteria, GEO production has been mainly attributed to planktonic and benthic aquatic strains and also to a few terrestrial filamentous and pseudo-filamentous taxa, which include nitrogen-fixers and non-fixers. More recently, however, Godoet al. [76] isolated from Lake Shinji, Japan, a strain of the colonial species *Coelosphaerium* sp. (Synechoccoccales) producing GEO. The axenic isolate was morphologically and molecularly (16sRna and 16–23sRna ITS analysis) characterized by the production of GEO, as determined in cultures [76]. These authors subsequently determined the presence of *geoA* in *Coelosphaerium* and assessed the presence of strains of this organism without *geoA* from the same lake, co-occurring at different stages of the blooms [77]. Indeed, *Coelosphaerium* sp. was also present in the lake when GEO off-flavor was not perceived. The authors consider this as an explanation for the lack of correspondence between GEO concentration and the number of *Coelosphaerium* cells.

MIB has been reported to be produced by several filamentous and two coccoid taxa, but to date, it has not been detected in nitrogen-fixing cyanobacteria. A few cyanobacteria (*Oscillatoria* spp.; *Phormidium limosum*; *Planktothrix* spp.; *Synechococcus* spp.; *Lyngbya bijugata* A4, from the soil) have been molecularly or chemically confirmed to produce both GEO and MIB [22,78,79]. Since the extensive review of Watson et al. [22], additional species and strains have been identified or confirmed to produce T&O compounds (Table 3). Out of 55 cyanobacteria investigated, 53 are filamentous, of the orders Nostocales and Oscillatoriales, 1 is from the order Synechococcales (*Coelosphaerium* sp. G2) [76], and 1 is from the Chroococcales order (*Microcystis* sp.) [80].

*Microcystis*, one of the most widely occurring bloom- and scum-forming aquatic cyanobacteria, produces multiple T&O compounds, including β-cyclocitral, dimethyl-disulfite, dimethyl-trisulfite, isopropylthiol, diisopropyl disulfide and diisopropyl trisulfide, although it has been thought not to produce GEO or MIB [22,92]. However, an axenic strain of *Microcystis* sp. isolated from a water reservoir in Southern Taiwan was found to produce both GEO and MIB [80]. Subsequently, Xuwei et al. [101] found a significant correlation between particulate MIB and *Microcystis* abundance in a non-blooming area of Lake Taihu (China). Even if there are only these sporadic indications, these findings suggest that at least some strains of *Microcystis* can produce these two T&O compounds.

There is debate about the relative importance of T&O producers other than cyanobacteria in freshwaters, with some evidence that *Streptomyces* can be an important T&O source in aquaculture plants, during run-off events, or along shorelines ([21] and references therein). A study encompassing various habitats, from aquatic to marginal zones in two drinking water reservoirs, found that a significant number of *Streptomyces* cells were at the vegetative stage in surface and bottom water, not only in the form of spores, suggesting their role as potential producers of GEO and MIB [102]. In the drinking water reservoir of Eagle Creek (IN, USA) with recurrent T&O events of GEO and MIB, Clercin and Druschel [103] analyzed samples collected monthly for a 1-year period to identify sources of T&O production, especially MIB, which often occurred when no, or scant, cyanobacteria were present. By 16S rRNA community analysis and subsequent sequencing, they identified potential producers among both cyanobacteria and actinomycetes. By Spearman correlation, it was possible to associate GEO with a small number of cyanobacterial taxa, especially *Planktothrix* sp. (*p* < 0.001), and MIB with *Streptomyces* (*p* < 0.001). The production of MIB by *Streptomyces* was stimulated by nutrient-rich inflows from the surrounding land following spring rains. Applying a complex metagenomic approach to samples from the same reservoir of Eagle Creek, Clercin et al. [104] confirmed a more important role of *Streptomyces* as a source of MIB, with the possibility of GEO production by actinobacteria and cyanobacteria, namely by *Streptomyces*, *Planktothrix* and *Nostoc punctiforme*.

The contribution of actinomycetes can also be significant in benthic cyanobacterial mats, whose role in the production of toxins and T&O compounds is becoming more evident. Gaget et al. [73] monitored the production of GEO and MIB and the composition of the bacterial community by next-generation sequencing for 1 year in two drinking water reservoirs in South Australia and found that benthic cyanobacteria could explain 10% of the variation in GEO concentration, versus 25% being attributed to actinomycetes. Expanding their analysis to different types of cyanobacterial mats in tropical, subtropical, and temperate regions (Australia, Southeast Asia and the United States), Gaget et al. [72] found cyanobacteria to be the sole producers of T&O more often than only actinobacteria (in 13%, versus 1% of cases respectively), with the majority of positive T&O samples being potentially sourced to both cyanobacteria and actinobacteria.

#### 2.2.2. β-Ionone and β-Cyclocitral

Other compounds potentially produced by cyanobacteria that are most frequently associated with T&O events are the nor-carotenoids β-ionone ((E)-4-(2,6,6-trimethyl-1-cyclohexen-1-yl)-3-buten-2-one) and β-cyclocitral (2,6,6-trimethylcyclohexene-1-carbaldehyde), also known as isocyclocitral (Figure 1) and some sulfur compounds, e.g., dimethyl sulfide (DMS) and dimethyl trisulfide (DMTS), that can be produced during active growth, with increased production stimulated by loss of cell integrity and/or cell death and the activation of catabolic enzymes [21,67]. However, since many sulfur compounds can also be the product of the anoxic decomposition of organic matter in sediments and do not represent a problem for drinking water since they are easily eliminated by conventional treatment [21], they will not be considered here.

The nor-carotenoids are produced by the oxidative cleavage of carotenes and xanthophylls. They play an important role in scavenging reactive oxygen species (ROS), protecting cells from oxidative damage, actively participate in light absorption and the protection of photosynthetic metabolites and functions, and as further roles, they can act as infochemicals and allelochemicals in deterring grazing ([105] and references therein). The most widely found nor-carotenoids are β-ionone and β-cyclocitral, the latter demonstrated to be produced only by *Microcystis* spp. among cyanobacteria [106] so far. Both β-ionone and β-cyclocitral have a lytic effect on cyanobacteria and algae. Occasionally, cyanobacterial lysis with the appearance of a bluish color has been observed in nature [107]: this phenomenon is essentially due to β-cyclocitral, whose oxidation to the corresponding acid reduces the pH of the medium leading to chlorophyll and β-carotene degradation. The cyanobacterial accessory photosynthetic pigment phycocyanin, whose color is usually masked in vivo by the other two pigments, is more resistant to low pH, and the cultures take the typical cyano-color [108,109]. On the contrary, β-ionone has an oxidative effect on all of the pigments and cultures are then discolored.

β-cyclocitral has a strong tobacco odor and is the only nor-carotenoid that can be identified as a primary odor source, most of the others contributing to the general bouquet of the specific nor-carotenoids. β-ionone has an odor of cedar wood, with some floral characteristics which, in very dilute alcoholic solution, resemble the odor of violets and roses. It is more common among various cyanobacteria and eukaryotes, and it is generated by carotene cleavage at different sites with respect to β-cyclocitral [22].

The enzymes responsible for oxidative cleavage are carotenoid cleavage dioxygenases (CCDs), which are highly species-specific and can be useful for tracking T&O-producers in blooms [22]. The activity of these CCDs is heightened by cyanobacterial cell rupture (e.g., by autolysis, cyanophage infection, grazing and water treatment processes) and large amounts of these compounds can be released. During an experiment to show the effect of released β-cyclocitral as a grazer-deterrent, Jüttner et al. [106] showed that *Microcystis* cells could release up to 77 attomol β-cyclocitral/cell after stimulation/rupture of cells by contact with *Daphnia magna*, while in the medium with undamaged cells, β-cyclocitral was almost undetectable. However, a recent study on the production phases of β-cyclocitral by an axenic *Microcystis* strain in a 9-week laboratory experiment showed a sharp increase in intracellular β-cyclocitral during the first log phase of growth, followed by a stable production during the stationary phase [110]. The condition of the cells was controlled, and the concentration was always related to intact cells in the system. Interestingly, the max. concentration reached during growth was 400 µg/L and 10.7 fg/cell, that is ~70 attomol/cell, the same concentration observed by Jüttner et al. [106].

### 2.3. Toxicity of T&O Compounds

#### 2.3.1. GEO and MIB

The OTC of GEO can be set between 1.3–1.0 ng/L [21,111]; MIB shows a similarly low OTC (6 ng/L) [111]. Few investigations have been made into the toxicology of GEO and MIB, and the current consensus is that these metabolites are nontoxic to humans via drinking water at environmentally relevant concentrations [64,112]. These conclusions mainly derive from earlier work showing that the concentrations at which GEO and MIB started to have adverse effects in aquatic organisms (e.g., cytotoxicity in rainbow trout hepatocytes; LC50 (Lethal Concentration), i.e., the concentration estimated to kill 50% of the animals, in sea urchin and algae; in vivo toxicity in the fish *Tanichthys albonubes*) are about three orders of magnitude higher than the human OTC and average environmental concentrations (nanomolar) (see [63]). Indeed, studies have detected MIB in raw water worldwide up to 700 ng/L [113,114,115,116,117,118]. Higher MIB concentrations were reported in a Brazilian Lake, of up to 1985 ng/L in raw water and 838 ng/L in treated water, during a summer bloom in 2004 [119]. To support the result reported in Brazil, assuming the highest intracellular concentrations of GEO and MIB reported so far, of 0.7 pg/cell and 0.14 pg/cell respectively [22], and a cyanobacterial population of up to 10^9^ cells/L in blooms and 10^12^ cells/L for scums, environmental concentrations of GEO and MIB up to the µM range are possible to achieve.

Regarding potential health effects in mammals, Bláha et al. [120] compared the toxicity of two different biofilms collected by manual scraping of the benthic biomass along the River Llobregat, Spain, in periods known to be essentially GEO-free (0 ng GEO/mg chlorophyll in the benthic mats and 16.1–20.3 ng GEO/L in water) or GEO-rich (0.34–1.45 ng GEO/mg chlorophyll and 61 and 110 ng GEO/L in water). MCs were not detectable in any sample. A set of in vitro toxicity tests was used as an indicator of different endpoints: (i) nonspecific cytotoxicity; (ii) hepatotoxicity; (iii) selective neurotoxicity; (iv) immunotoxicity; and (v) mutagenicity, by using mouse-derived organo-specific immortalized cell lines, which unfortunately are not the best model to extrapolate results to actual effects in humans. However, very weak and scattered effects were reported, independently of the presence of GEO [120]. Burgos et al. [119] tested MIB toxicity in vitro using Chinese Hamster ovary cells with the Comet assay (7.5, 15, 30 and 60 µg MIB/mL) and in vivo with *Drosophila melanogaster* as test systems to detect gene and chromosome mutations or mitotic recombination (125, 250 and 500 µg MIB/mL). Despite the high concentrations tested with respect to environmental concentrations, no genotoxicity effects were reported, with the exception of some positive findings in the Comet assay on Chinese Hamster ovary cells at the highest concentration tested [119]. This lack of genotoxic potential is consistent with previous results on *Salmonella typhimurium* (with and without metabolic activation) testing up to concentrations reaching cytotoxic levels, approximately six orders of magnitude greater than the odor threshold concentrations in the ng/L range [121]. When GEO (12.5, 25, 50, and 75 μg/mL) and MIB (12.5, 25, 50, 75 and 100 μg/mL) were tested on a HepG2 cell line, the results showed that at the highest concentration tested, both compounds were cytotoxic but unable to induce either DNA damage (thus confirming, again, no genotoxic potential), or events associated with chromosomal instability, or alteration in the expression of some genes [122]. A wider range of concentrations (10 ng/L−300 mg/L) was tested on human-, monkey- and dog-derived cell-immortalized lines: most of the concentrations used were high when compared with environmental concentrations (which are in the high nanomolar range up to the low micromolar range, as above), but despite this, no cytotoxic or other effects were found [116].

Zhou et al. [19] tested the effects of a range of concentrations of GEO (50, 500, 5000 ng/L), including some environmentally relevant dosages on developing zebrafish (*Danio rerio*) embryos from 2 to 96 h post-fertilization. No effects were observed on hatchability, malformation or mortality. However, body length increased, and genes related to growth and thyroid functioning were upregulated in a dose-dependent manner, starting from the mid-dose, and markers of oxidative stress were reported. In line with this last finding, the highest concentrations induced cell apoptosis and a high level of oxidative stress. Since it was reported that at 12 h, the volatility of GEO concentrations (50, 500 and 5000 ng/L) was, as expected, dependent on the initial concentration in water (i.e., 58%, 75% and 85% respectively), the actual exposure of the test system did not vary over time proportionally with respect to the initial nominal concentration [19]. In addition, it was clearly found again that the solvent used to dissolve the test item can strongly affect the obtained results. This highlights the need to measure the actual exposure of the test organism also in vitro instead of the nominal concentration and to follow the biokinetics of the test item [123] to exclude the possibility that toxicity tests, as done previously, could have resulted in effects at concentrations lower than the nominal ones. Zhou et al. [18] later tested MIB at 0, 0.5, 5 and 42.8 μg/L on zebrafish embryos. They observed similar results of increased body length, the presence of oxidative stress and apoptosis and upregulation of genes involved in growth and thyroid development.

In terms of GEO and MIB terpenoids, data on their toxicological properties are very scant and have been mainly obtained with aquatic organisms. One investigated endpoint giving consistent results in different test systems and methods is the lack of genotoxic potential. When cells of mammalian origin were used in vitro to check for end-points other than genotoxicity, their relevance was limited since immortalized cell lines (which are in some cases transformed) were used, which are known to have an altered system for metabolizing xenobiotics, including GEO and MIB. Overall results appear to indicate a low potential for inducing significant health effects in vivo. However, most of the available results are affected by experimental design, not considering the actual exposure conditions of the test system and using poorly suitable experimental models. They are not, therefore, useful to extrapolate the observed effects (if any) to humans. The fact that the most recent papers use zebrafish embryos, an experimental model which is increasingly considered a good early marker of potential effects in mammals [124,125], suggests possible subtle and specific effects, and indicates that more high-quality, relevant studies should be carried out to elucidate the kinetics and toxicological potential of GEO and MIB.

#### 2.3.2. β-Ionone and β-Cyclocitral

β-ionone is naturally found in many plant essential oils (e.g., rose and violet oils). However, it is also industrially produced on a large scale for a variety of uses [126], including as a fragrance in toiletry products and as a flavoring compound in food, where it has been found at concentrations ranging from 0.5 to 10 mg/L [127]. Lalko et al. [128] reviewed the use of β-ionone as a fragrance in cosmetics. For use as a food flavoring, the International FAO/WHO Joint Committee on Food Additives (JECFA) has evaluated its toxicity, reporting that: (1) β-ionone is absorbed after oral exposure and metabolized in the liver to polar metabolites and excreted in the urine unchanged or conjugated with glucuronic acid: (2) a very low acute toxicity occurs via the oral route. In addition, on the basis of a sub-chronic (90 days) oral (diet) toxicity study in rats, a NOAEL (No Observed Adverse Effects Level) (i.e., the highest concentration used in a toxicity testing at which no adverse effect have been observed, generally used as a threshold for toxicity) of 1000 ppm (72 and 83 mg/kg bw/d, for males and females, respectively) was derived based on liver effects (increase in γ-glutamyl transferases as a marker of liver diseases and liver cell hypertrophy), establishing an Acceptable Daily Intake (ADI) (i.e., the dose that could be ingested daily for the entire life without experiencing any significant adverse effect) of about 0.1 mg/kg body wt for α- and β-ionone individually or in combination [129]. β-ionone shows no genotoxic potential based on in vitro *Salmonella* mutagenicity assays with and without metabolic activation and an in vivo mouse bone marrow micronucleus assay (750 mg/kg bw, intraperitoneal) [127,130,131]. In the subchronic study evaluated by WHO to derive the ADI, no histopathology changes in male and female rat reproductive organs, and no alterations in sperm counts, motility and morphology, or of estrous cycling were reported. Consistent with this observation, a more recent study on the developmental toxicity of β-ionone (0, 125, 250, 500 and 1000 mg/kg body weight/day) administered orally to rats on days 6–15 of gestation (GD6-15), and on the embryotoxicity of a single oral dose of β-ionone (1000 mg/kg bw) given on GD11, indicated that the NOAEL for maternal and developmental toxicity was 500 mg/kg bw per day. No embryotoxic effect over the dose range tested was observed except for a higher embryo-lethality secondary to overt maternal toxicity [126]. The derived NOAEL was slightly higher but was similar to NOAELs reported by the OECD [127]. On the contrary, β-ionone has shown some bioactivity against *D. rerio* embryos [20]: the 96 h LC50 of β-ionone to zebrafish was 1321 μg/L, and exposure to higher concentrations caused changes in embryonic development, including a decreased hatching rate, morphological abnormalities and an increase in mortality rate, plus significant decreases in locomotor activity and catecholamine neurotransmitters levels. It should be noted that the effects observed at highly toxic doses (higher than LC50) are very likely secondary to the generally poor health status of the test system at those high doses. In addition, differences in sensitivity to potential toxicants might be due to species specificity [132] and to the different kinetics in the models used.

The estimated OTC of β-ionone is 7 ng/L, which aims to prevent exposure via drinking water being more than two orders of magnitude lower than the acceptable daily intake; therefore, it does not represent a health risk via this route. In agreement, the FDA considers β-ionone to be Generally Recognized as a Safe (GRAS, 21 CFR 172.515) chemical for its intended use in food. It is also included in the list of flavorings authorized by the EU according to the evaluation carried out by EFSA [133]. Regarding use in feed, β-ionone is considered safe at the normal use level (5 mg/kg complete feed) for salmonids, veal calves and dogs, whereas for the remaining target species, the use level of 1 mg/kg complete feed is considered safe [134]. In addition, due to its relatively low toxicity and use as a flavoring substance and as an insect attractant or repellent, β-ionone is receiving increasing attention from the biomedical community since antibacterial, fungicidal and potential antitumoral properties have been claimed [135]. It is particularly notable that a single oral dose of 100, 300 and 600 mg/kg to rats induces the activity of CYP2B1 in the liver [136], which can be the cause of potential interaction with other chemicals biotransformed by the same enzyme.

Furthermore, in the European Chemical Agency (ECHA) public database, >98% of the registrants have classified β-ionone as Aquatic Chronic 2 (https://echa.europa.eu/information-on-chemicals/cl-inventory-database/-/discli/details/25343 (accessed on 30 January 2023)), with the H phrase H411: toxic to aquatic life with long-lasting effects. Some studies have been published on the effects on algae and aquatic organisms. For example, Du et al. [137] observed a positive relationship between growth and β-cyclocitral and β-ionone concentrations in the aquatic macrophyte *Lemna turionifera*, including decreases in chlorophyll content and photosynthetic O_2_ production and a downregulation of genes involved in growth.

β–cyclocitral (or isocyclocitral) has been identified as produced exclusively by *Microcystis*. By consulting ECHA public information (https://echa.europa.eu/information-on-chemicals/cl-inventory-database/-/discli/details/29773 (accessed on 30 January 2023)), it is evident that there is no harmonized classification of this compound. More than 85% of the registrants indicated by auto-classifying the chemical that after a single exposure it is acutely harmful if swallowed (H302), in contact with skin (H312), or if inhaled (H332). All of the registrants (100%) classified it as a skin irritant (H315) that causes serious eye irritation (H319) and as a respiratory irritant (H335) according to the EU Regulation known as CLP (classification, labeling and packaging of chemicals) [138]. However, it should be considered that the CLP classification is exclusively hazard-based and that no other information about risks is available, especially after repeated exposure. Isocyclocitral is also included in the list of flavorings authorized by the EU, according to the evaluation carried out by the EFSA [133], in which it is reported that, based on chemical structure, the compound is expected to be oxidized to the corresponding carboxylic acid, conjugated with glucuronic acid and excreted as urinary metabolites. Since apart from an acute toxicity study, cited in the ECHA dossier as grey literature but not available (Registration Dossier—ECHA (europa.eu)), no further data have been produced. The evaluation of β-cyclocitral toxicity has been carried out by applying the Threshold of Toxicological Concern (TTC) approach [139]: β–cyclocitral belongs to Cramer Class I (characterized by low toxicity) and therefore, levels below the threshold of 1800 µg/person per day are expected not to induce any relevant health effect in an adult of 60 Kg bw [133]. This was also the approach followed by RIVM, evaluating isocyclocitral when used as a fragrance [140] Consequently, the identification of the level of exposure is crucial to understand the toxicity of β-cyclocitral, produced in water.

β-cyclocitral is present at extremely low concentrations (e.g., 2.6 attomol/cell) in live *Microcystis* cells, but since a cell rupture activates a rapid carotene oxygenase reaction, higher amounts (e.g., 77 ± 5.5 attomol β-cyclocitral/cell) can be produced [106]. Assuming that, during a bloom, the presence of around 10^9^ cells/L can commonly occur, then about 12 µg/L can be present in dissolved form in the water after cell rupture (e.g., in senescent blooms). Other laboratory studies have determined much higher and highly variable intracellular concentrations of β-cyclocitral in live cyanobacterial cells (from 10 to 865 fg/cell) [110,141], corresponding to 400–2000 µg/L and lower environmental concentrations of ~700 µg/L with an intracellular quota of 54–145 fg/cell [141]. Considering the difficulties in removing T&O compounds from the water via traditional water treatment processes, the consumption of 2 L of drinking water per day is expected to be lower than the TTC for β-cyclocitral after oral exposure or higher than that only during sporadic events. However, the potential for irritation suggested by the auto-classification and reported by ECHA should be further investigated, especially when this compound is inhaled, even if its OTC of 19.3 µg/L [67] suggests that the concentration at which it is perceived is still much lower than its TTC.

β-cyclocitral also has ecotoxicological effects. Sun et al. [142], in an experiment on the toxicity of β-cyclocitral to microalgae, followed a series of parameters, including photosynthetic pigment content, which were gradually reduced, and the efficiency of photosynthesis through Fv/Fm, which gradually declined, indicating that β-cyclocitral can kill the alga *Chlamydomonas reinhardtii* by inducing apoptosis. At concentrations > 0.05 mM β-cyclocitral decreased the algal growth rate, and at a concentration of 0.4 mM, it activated the upregulation of apoptosis genes, with oxidative stress and death of the whole algal population within 24 h.

In summary, it seems that no major human health effects can be attributed to the production of β-ionone, for which some toxicological data are available, enough to derive a TDI. It must be stressed, however, that TDI is derived to protect from long-term to lifetime consumption and may be considerably lower than daily doses considered ‘safe’ for short-term consumption as it could be for many T&O compounds considering the intermittence and duration of cyanobacterial blooms. On the contrary, very scant data are available for β-cyclocitral—if it is expected that no adverse effect can be associated with the consumption of drinking water, then effects related to inhalation deserve further investigation.

## 3. Environmental Evidence of Co-Occurrence of T&O and CTX

A systematic literature search combining the strings “cyanotox*” and “taste and odor” with the boolean operator AND in the fields “Title/Abstract” and “all fields” was performed to look for studies that dealt at the same time with CTX and T&O, either in field or laboratory studies, including by molecular analysis. PubMed, Scopus, Science Direct and Web of Science were consulted. The search resulted in the 20 studies reported in the following sections (Section 3 and Section 4) (Table 3) after deleting non-pertinent papers.

A total of 12 environmental studies analyzed the co-occurrence of T&O and CTX so far (Table 3): despite available results suggesting that this is a frequent event, no direct relationships have been identified between these groups of metabolites and producing organisms or even between the different types of these compounds.

Zamyadi et al. [14] collected data from 21 water treatment plants (WTP) and found that at 20 out of 25 sites, at least 1 cyanotoxin (usually CYN) co-occurred with both GEO and MIB. Similarly, Graham et al. [99] found a co-occurrence of various CTXs (MCs, CYN, STX and ATX) and T&O compounds (GEO and MIB) in 23 lakes from the Midwestern United States on a single sampling date in late summer. Communities were dominated by clusters of *Anabaena*, *Aphanizomenon*, and/or *Microcystis* in 57% of the blooms, followed by *Microcystis* (17%) and *Cylindrospermopsis* (17%) alone. CTX and T&O compounds co-occurred in 91% of blooms, and more specifically: MCs (max 19,000 µg/L) co-occurred with GEO (max 0.86 µg/L) in 87% of blooms and with MIB (max 0.06 µg/L) in 39%, whereas ATX (max 9.5 µg/L) always co-occurred with GEO, and only in 43% of blooms with MIB [99]. No correlations between the different variables and potential CTX- and T&O-producers was found, and some of these secondary metabolites were also detected when no known potential cyanobacterial-producers were present. Conversely, many potential CTX- and T&O-producers were dominant in samples with no detectable metabolites. However, this apparent anomaly is not surprising since T&O compounds can also be produced by yet-unknown sources, as demonstrated by Otten et al. [97] using shotgun metagenomic analysis. In addition, producer-cell population peaks do not necessarily correspond with T&O or CTX maxima, as shown by Li et al. [94], who followed a bloom for two months in the Yanghe Reservoir, northern China, with daily sampling. The dominant cyanobacterium *Anabaena spiroides*, a known GEO-producer, reached a peak of 7·10^4^ cells/mL, followed by *Microcystis* spp. (including *M. aeruginosa*, *M. wesenbergii*, *M*. *novacekii*, and *M. botrys*), with a peak of about 3·10^4^ cells/mL. During the bloom, intracellular concentrations of GEO (>85% of total GEO) reached a very high value of ~7 µg/L with a 1–2-day lag with respect to the population density of *A. spiroides*. Nevertheless, in this specific case, *Anabaena* cells and GEO still strongly correlated (R^2^ = 0.92), and the calculated cell quota was 0.1 pg/cell. Among the CTX, the ATX concentrations were much lower than MCs (<1 µg/L), and MC-RR (the most abundant of three MC variants) reached intra- and extra-cellular maxima of 80 µg/L and 15 µg/L, respectively. At variance with GEO and *Anabaena*, correlations between MCs and their potential producers (*Microcystis* spp. and *Anabaena*) were not so strong. Li et al. [94] concluded that it is necessary to measure the actual metabolite concentrations and not to rely upon the population size of the potential T&O- and CTX-producers, with the further need to localize the metabolites (i.e., intra- and/or extracellular pool sizes) to apply the most adequate actions for effective water treatment plant operation. Other studies confirm that predicting either T&O or CTX concentrations simply from the abundance of potential producers is unreliable [98,143,144].

Gaget et al. [72] compared the production of T&O compounds and CTX by benthic cyanobacterial mats in temperate, subtropical and tropical regions and found that MIB production was higher in warmer climates, as was the case for ATX, STX, CYN and their genes. MCs and the MC-biosynthesis gene *mcyE* were the most abundant by 2–3 orders of magnitude and were highest in temperate areas. The distribution of GEO was not related to any specific climate area, and often, cyanobacteria were the only known sources of T&O.

In Lake Taihu, a large freshwater shallow lake (2338 km^2^) in China, supplying water for domestic, industrial and agricultural use for approximately 10 million residents, cyanobacterial blooms have proliferated since the 1980s, especially *Microcystis* spp. Here, among the T&O compounds co-occurring with CTX, β-ionone and β-cyclocitral have been frequently found. In Taihu’s Gonghu Bay, during a systematic seasonal and spatial monitoring campaign in 2008, several T&O metabolites, namely some sulfur compounds, β-ionone, β-cyclocitral, GEO and MIB, were found co-occurring with MCs. However, a significant correlation has been found only between intra- and extra-cellular MC concentrations (max 35 µg/L and 0.46 µg/L, respectively) versus intra-cellular β-ionone and β-cyclocitral concentrations (50.55 ng/L and 537.61 ng/L), that were higher and more frequent than other T&O compounds measured in the lake [93].

In another study on the whole of Lake Taihu, the lake was divided into two areas according to *Microcystis* (the dominant cyanobacterium) population density (blooming and non-blooming area: *Microcystis* > or < 1.5 × 10^7^ cells/L). Intra-cellular β-ionone, β-cyclocitral and MIB (max monthly average 83.54, 354.96 and 15.95 ng/L respectively) were detected during the *Microcystis* bloom in the blooming area and were correlated with *Microcystis* cell population size. The highest extracellular fraction of MIB (87.06 ng/L) was not correlated to any variable. In the non-blooming area, only MIB was found at high concentrations (intra-MIB 115.59 and extra-MIB 82.70 ng/L), showing a strong correlation with *Oscillatoria* population size [101]. In a more recent survey on the whole lake (from May 2019 to Feb 2020), Li et al. [95] identified four T&O compounds (GEO, MIB, β-cyclocitral, β-ionone) and three CTX classes: MCs, CYNs and STXs. These authors found a significant and positive linear relationship between total MCs and β-cyclocitral or β-ionone concentrations (*p* < 0.001). Further, total CYNs correlated significantly with MIB (*p* < 0.05). The concentrations of CTX were lower than previously detected in Lake Taihu, being 2 µg/L MCs and 0.6 µg/L CYNs, while the concentration of β-cyclocitral was comparable with other findings, being around 240 ng/L.

Shang et al. [13] analyzed the eastern drinking water source at Lake Chaohu (China) to determine the efficiency of the full-scale drinking water treatment plant and found co-occurrence of three MC variants (MC-LR, -YR and -RR) and MIB, β-ionone and β-cyclocitral, but no simple relationships between their concentrations or between these compounds and *Dolichospermum* and *Microcystis* population densities, the dominant cyanobacterial species in spring and summer-autumn, respectively. The highest concentrations of the compounds were found in different seasons, with GEO and MIB being mostly in the dissolved fraction (MCs 4.9 µg/L; GEO and MIB 17.5 and 72.8 ng/L; β-cyclocitral and β-ionone 53.1 and 64.2 ng/L).

Yen et al. [100] determined the concentration of MIB and MC in two eutrophic reservoirs in Taiwan, with 22 environmental variables over 1.5 years and proposed a model to predict the two compounds. Temperature alone could explain about 50% of the variations in MC and MIB concentrations, which reached their maxima of about 300 and 15 ng/L, respectively, in summer.

In summary, some environmental studies, including T&O compounds as a variable, are available from recent years, but the number is extremely scant when compared with the still-increasing number of publications on CTX alone. In addition, the analyzed data show that it is not currently possible to find any specific correlations between either environmental CTX or T&O concentrations versus cyanobacterial population sizes. This could be due to the confounding factor of T&O biosynthesis by bacteria (e.g., actinobacteria) rather than cyanobacteria, which can also be massively present during blooms. In addition, the situation is likely to be influenced by the particular cyanobacterial species present, their genetic characteristics (Section 4) and the particular CTX and T&O compounds under investigation, as well as the environmental conditions (climate, nutrients, etc.) in the waterbody and its catchment.

## 4. Molecular Studies: T&O and CTX Producers

Early data on species thought to produce both T&O and CTX as reported by Watson [63] came from different studies: when a species was recognized as producing T&O and was also known to be producing CTX, it was expected (but not necessarily experimentally verified) that it could produce both. As reviewed in Section 2, there are often individuals in the same population that possess the genes for CTX or T&O biosynthesis (but do not always express them) and others that do not have the genes. Despite the environmental and water management value of a genetic understanding of the potential production of CTX and T&O compounds, the genetic potential to produce both classes of compounds at the same time has not been adequately investigated in either isolated strains of cyanobacteria or in the field. The few investigations to date indicate that there are relatively few strains that can produce both CTX and T&O compounds. Suurnäkki et al. [78] found that 6 out of 21 GEO-producers (over 100 strains analyzed), identified by the occurrence of GEO and the presence of the GEO gene *geoA*, were also ATX-a and MC-producers (Table 3). Among the 15 strains not producing detectable CTX, only 2 produced GEO and MIB at the same time. Otten et al. [97] used shotgun metagenomic analysis to identify the producers of MCs and T&O compounds in the Cheney Reservoir (Wichita, KS, USA), which has had T&O and MC events regularly since the 1990s. They identified *Anabaena* as the main GEO producer and *Microcystis* as the MC producer. MIB was only detected in a few samples, with benthic *Oscillatoria limosa* tentatively as one of the T&O-producing strains in the reservoir. 

In a detailed phylogenetic analysis of genomes from the literature and newly sequenced genomes, Driscoll et al. [84] aimed at a better understanding of the relationships among Nostocales cyanobacterial blooms. The 15 sequences analyzed formed a separate clade (ADA from the genera of *Anabaena*/*Dolichospermum*/*Aphanizomenon* groups) among the Nostocales, with four species-level subgroups. Numerous genes involved in nutrient acquisition, and metabolic and physiological traits were identified that could influence niche partitioning. All the species of the ADA clade potentially produced bacteriocins, a large group of bioactive peptides; only four potentially produced GEO, all of which also had, in various combinations, CTX genes other than bacteriocins; three potentially produced aeruginosin, one STX and one anabaenopeptins. In a subsequent study, the same authors added 16 more species to the ADA clade, mostly from water blooms and enlarged the phylogenetic analysis to other strains sequences from a database for a total of 43 strains [85]. They repeated the analysis as in Driscoll et al. [84] but found only one more strain potentially producing GEO and anabenopeptin.

Kim et al. [81] isolated non-axenic strains of cyanobacteria from a river in South Korea and determined the presence of *mcyA* (for MCs production), *gys* (for GEO) and *mibC* (for MIB) genes, and investigated the effects of temperature on MCs, GEO and MIB production. Of the seven species investigated, two strains of *Dolichospermum circinalis*, one coiled and one straight, *Aphanizomenon flos-aquae*, *Microcystis aeruginosa*, *Oscillatoria limosa*, *Planktothricoides raciborskii* and *Pseudoanabaena mucicola*, only the coiled strain of *D. circinalis* had at the same time the *gys* and *mcyA* genes.

A more prominent role for benthic cyanobacteria and, more specifically, for benthic cyanobacterial mats is becoming evident in the production of CTX and T&O compounds by the same source. Gaget et al. [90] isolated many strains of benthic cyanobacteria from two South Australian drinking water reservoirs. They confirmed the toxicity of the strains producing MCs and CYN, also looking at the presence of the associated genes. Out of 23 isolates, only *Phormidium ambiguum* strain AWQC-PHO021 produced intracellular CYN and deo-CYN (739 ng/mg dw and 107 ng/mg dw, respectively) [90] together with 37.9 µg extracellular GEO/L [73]. In their subsequent study on different climatic areas, 2 out of 20 Oscillatoriales strains from cyanobacterial mats were found to produce GEO and ATX-a and 2 produced MIB and ATX [72] (Table 3).

Taken together, of all the hundred cyanobacterial species/strains analyzed in these studies, about 40% potentially produce GEO or MIB and only 15% can potentially simultaneously produce T&O and CTX, indicating that for much of the time, the production of T&O and CTX is probably due to different taxa or strains of cyanobacteria, and/or non-cyanobacterial species.

## 5. Environmental Roles and Parameters Affecting T&O and CTX Production

While numerous advances in the genetic, molecular and toxicological characteristics of CTX have been made over recent years, lesser understanding exists of their natural functions. Original functions, including quorum sensing and cell communication within and between cyanobacteria and with other microbes, may be distinguished from secondary benefits, e.g., as deterrents against eukaryote microbial- and invertebrate-grazers [145]. In addition to some CTX, T&O compounds also appear to act as quorum-sensing molecules towards cells of the same species [146] and as allelopathic or attractant compounds versus different species, either competitors and/or predators [106,147]. However, as with CTX, the full range of T&O compound functions is not fully clarified. Not knowing their exact specific role(s), it is also difficult to predict or identify all of the possible parameters regulating their biochemical formation, which are under the control of multiple factors from the external environment (e.g., nutrient concentrations and speciation, temperature, light, competition) and internal signaling (e.g., oxidative stress, changes in cellular metabolic activity) as indicated in earlier reviews (e.g., [5,22,25,148,149]), with regulatory factors often acting in combination or differently depending on the producer-species. It can therefore be expected that laboratory experiments, testing one or few parameters at a time using isolated monoclonal cyanobacterial strains, may give contrasting results versus field studies with natural populations and can provide a mixed and only partial, inconclusive understanding. It does not seem currently possible, therefore, to establish general trends/correlations as a possible input for predictive modeling. Here, only the most recent papers are discussed to indicate the complexity of the relationships between T&O metabolites and the environment.

In the cyanobacteria *Microcoleus vaginatum* and *Phormidium retzii*, respectively, MIB- and GEO-producers, temperature and light optima for growth are also optimal for the production of T&O [87]. However, this is not a general rule: indeed, for the cyanobacteria *Pseudanabaena* sp. and *Anabaena ucrainica*, producers of MIB and GEO, the temperature and photosynthetic irradiance at which the cells grow optimally inhibit the production of the two VOCs and when growth was inhibited the production of GEO and MIB increases [150]. In *Oscillatoria*, growth and GEO production respond to temperature in the same way, while photosynthetic irradiance acts in the opposite direction [89]. The effects of different irradiances have been studied on the growth and MIB production of *Planktothrix* sp. FACHB-1375 [83]. Within a large range of irradiance (5 to 250 μmol photons m^−2^ s^−1^), growth was maximal at intermediate values and slightly lower at the highest irradiance, while MIB production was highest at the maximum growth rate and decreased dramatically at the other irradiances. Therefore, the effect of temperature and light highly depends on the cyanobacterial species and growth phase.

Other papers also show the effects of light, temperature and nutrients on the ratio between intra and extracellular T&O compounds. Lu et al. [88] assessed the production of MIB by *Planktothricoides raciborskii*, isolated from a low-latitude water reservoir, and showed in a growth factorial experiment that the only significant effect on MIB production was due to temperature. In addition, extracellular MIB concentration was highest for the high-temperature set (75.7 ± 11.5%). Even if not significant, the extracellular MIB ratio increased from 37.3 ± 4.2% to 57.3 ± 8.9% with the increase in irradiance from 13.5 to 81 μmol photons m^−2^⋅s^−1^. However, this interdependence has not been investigated with other cyanobacterial species so far, and no wider conclusions can be drawn.

In laboratory experiments, Oh et al. [82] determined that an increase in GEO concentration in systems with high N content (250 mg-N/L vs. 2.5 and 0 mg-N/L) was indirectly due to an increase in population density in two species of *Anabaena* (NIER and Chusori) isolated from a Korean river and lake. Further complex relationships between the production of GEO and light, temperature, N (nitrate and ammonium) and P were observed in *Anabaena* sp. [151]. Interestingly, the conditions that enhanced the production of chlorophyll a decreased the production of GEO and vice versa. Perkins et al. [152], in analysis of a one-year sampling and historical database from a UK drinking water reservoir, found that the concentrations of MIB and GEO strictly correlated with ammonium concentration and with no other form of N, as long as P was not growth-limiting.

Environmental conditions not only affect the concentration and the amount of volatile T&O production by microbes and cyanobacteria specifically but also alter the relative concentrations of the different products and the whole toxicity versus other algae. Xu et al. [153] showed a differential toxicity versus the green alga *Chlorella vulgaris* of the T&O produced under different sources of nitrogen by *Microcystis flos-aquae*: T&O production under non-N conditions was quantitatively higher and more toxic to *Chlorella*. Similarly, when *M. flos-aquae* was grown under different P forms and limiting P concentration [154], the level of T&O produced was different and more toxic to *Chlamydomonas reinhardtii* under P-limiting growth conditions.

Since both terpenes and MCs can have a role in protecting cyanobacterial producer-cells from oxidative stress, it may not be necessary or convenient for cells to produce both classes of metabolites at the same time or at the same rate. Environmental investigation of these possibilities may provide some indications of the relationships between CTX and T&O compounds in the same species, although no such studies are known.

Zhang et al. [92] investigated the effect of co-culturing on the production of T&O compounds using variable relative cell population densities (1:1, 2:1, 1:2) of two non-axenic cultures: MC-producing *Microcystis aeruginosa* FACHB-905 and non-MC-producing *Pseudanabaena* sp. FACHB-1277, producers of β-cyclocitral and MIB, respectively. Cell population density and the production of intra and extracellular T&O compounds were investigated. In the co-cultures, *M. aeruginosa* population density was always higher than in the control, while *Pseudanabaena* sp. was higher only at the ratio 1:1 and was lower in the other systems. The authors also observed an increase in the extracellular and total concentrations of the T&O compounds in both species. With *Pseudanabaena*, the amount of extracellular MIB at the end of the experiment could be related to senescent cell abundance, while with *M. aeruginosa* the cells were still actively growing, suggesting an active transport outside the cell of β-cyclocitral. Zhang et al. [92] further measured the growth of each strain in the spent medium of the other strain. *M. aeruginosa* growth was stimulated by the spent medium of *Pseudanabaena* sp., while the reverse was not true. The total production of β-cyclocitral and MIB followed the same trend of the density of the two strains, and there was no increase in extracellular MCs in the systems with *M. aeruginosa*.

Since T&O compounds can lyse cyanobacterial cells and can also induce oxidative stress on cyanobacteria [155], it could be hypothesized that MCs, by protecting the cells from oxidation, could confer a competitive advantage upon the MC-producing species. This would have important implications in the development of a toxic bloom in the presence of T&O compounds.

The same complex relationships apply to CTX, and many important factors have been highlighted, especially for the most studied class, i.e., MCs [5,15,156]. Multiple roles of MCs have now been recognized and accepted. As for T&O, the toxins can be used as allelopathic effectors, or used for quorum sensing within the same species; they can protect cells from oxidative stress and contribute to photosynthesis and light adaptation, can be involved in iron acquisition, nutrient metabolism and storage, and in colony formation. Two recent papers provided further confirmation of MCs’ role as density-dependent infochemicals: they can upregulate the expression of *mcy* synthase [157] and can stimulate the intracellular MC binding to the primary enzyme of cyanobacterial CO_2_ fixation, RubisCO, and finally, induce RubisCO relocation from the cytoplasm to the periphery of the cell [158]. However, information on the ecological role(s) and parameters affecting other CTX classes is lacking.

## 6. Conclusions and Gaps in Knowledge

Since several T&O compounds are produced by bloom-forming cyanobacteria, including toxigenic species, it has often been questioned whether, and in some cases assumed, that T&O production is associated with the presence, or potential presence, of CTXs in water resources [10,21,64,67]. For example, during a *Phormidium* out-growth in South Australian water reservoirs, the presence of MIB/GEO was used as a surrogate test for CTX occurrence in the water supply [159]. However, increasing investigations indicate that this potential relationship is not consistent. As pointed out, e.g., by Watson & Jüttner [21], not all cyanobacteria produce T&O compounds; the latter are also produced by non-cyanobacteria, and often T&O production is uncoordinated with CTX production. The use of T&O compounds as a surrogate, or warning signal of a toxic bloom, should not be considered a simple, reliable correlation but should take into account the fact that the production of T&O compounds is the result of interactions between the physiology of organisms, large-scale ecological events and food web relations. A polyphasic approach is needed [22] to better understand the biological roles of T&O compounds and under what conditions they can contribute in a predictive manner to water quality and safety assessment. By producing T&O, other organisms present in water and often associated with cyanobacterial blooms can act as confounding factors, and unfortunately, these sources appear to be often ignored in the studies on T&O and cyanobacteria. To support this, the few data available show that only ~15% of the analyzed cyanobacterial species possess the genes for GEO or MIB plus some genes for CTX. In addition, the environmental studies on their co-occurrence, as reported in this review (Section 3) and considered by Zamyadi et al. [14], are scant. The latter collected many published studies on CTX- and T&O-producing blooms since the 1990s, but only two investigations [99,100] have systematically analyzed the co-occurrence of CTX and T&O compounds on a large scale. Otherwise, data were obtained from studies that either investigated CTX or T&O. With so little data available, it is impossible to understand if and how the two groups of metabolites can co-vary or influence each other. For these reasons, using T&Os as early warning surrogates for CTX is not currently valid.

Regarding the possible triggers for T&O or CTX production, it is not currently possible to identify any specific or general rules correlating environmental conditions (or the biochemical status of the cyanobacterial potential-producer cells) to predict whether and to what extent a bloom can be producing both CTXs and T&O compounds. The interrelationships among all of the variables are complex and mostly unknown, and the available data indicate that it is strictly dependent on the producer species. Furthermore, waterbody management actions on a global scale, including P reduction, seem not to be the solution for events that are tightly connected to local environmental conditions, to the specificity of the habitat and to the complex relationships with other species [62].

The known CTX of primary concern as human health hazards, including MC, NOD, CYN, STX, ATX and GNTX, are recognized to be toxic to humans, domestic animals and some environmentally relevant species, at relatively low-, but environmentally encountered, concentrations. However, the available toxicity data for those T&O compounds most frequently produced by cyanobacteria and associated with bloom episodes (namely GEO, MIB, β-cyclocitral and β-ionone) suggest that at environmentally relevant concentrations, systemic human health effects due to T&O compounds exposure are not currently envisaged (based on the limited available data). This is true, also considering that the concentrations at which they can be detected by smell and taste, thus potentially avoiding further exposure, are greatly lower than what is expected to represent a health risk, with a safety margin that can also include saturation of odor receptors, giving rise to habituation to the smell or taste. In this respect, it is also important to consider the relationship between smell (or taste) intensity and the concentrations of the compounds that can be described by various mathematical models [160]. Indeed, in some cases, the relationship is very steep, meaning that low increases in concentration correspond to significant increases in the intensity of human sensing, whereas for other T&O compounds, the slope is very low, and very little change in the intensity corresponds to large changes in concentration. This indicates that the perception and intensity of a taste or odor cannot always be indicative of a low concentration of compounds and that the concentration/intensity relationship should be known.

Several knowledge gaps still exist in the toxicity assessment of T&O compounds, even as single chemicals. In addition, the effects of combined exposure to defined mixtures of CTX and T&O compounds and to combinations of co-occurring T&O compounds require further toxicity assessment. The health significance of the co-occurrence of T&O metabolites with environmental toxicants other than CTX has not been investigated to assess if this scenario can represent an emerging risk for human, animal or environmental health from a One Health perspective. The data available are not sufficiently robust (especially regarding the environmental validity of the exposure data) to allow such assessment, considering the simple interaction due to the co-presence of multiple T&O or to CTX and T&O. However, additive toxicity due to multiple exposures can be assumed, following the most recent procedures to evaluate mixture toxicity [139,161]. This could be the case for T&O compounds and CTX sharing similar effects or mechanisms of action, e.g., causing oxidative stress, such as GEO and MCs [162] or CYN [163]. Indeed, at CTX and T&O environmental concentrations, synergistic toxicity (which from a health protection perspective is the worst scenario) is not expected, being the concentrations, at least for T&O, well below the toxicity threshold [164]. 

Another point to consider regarding the co-occurrence of different T&O is the possibility that interactions among odor-producing substances can result in a strong increase in the odor thresholds. Examinations of such mixtures have provided preliminary indications that although the intensities of compounds with a similar aroma can be additive, the intensity of the mixture is usually lower than the sum of the individual intensities [165]. However, for substances that clearly differ in their characteristic aromas, the odor profile of a mixture is composed of the odor profiles of the components added together only when the odor intensities are approximately equal [165]. If the concentration ratio is such that the odor intensity of one component predominates, this component then, largely or completely, determines the odor profile. The toxicological impacts of co-exposure to CTX plus inorganic or anthropological agents/pollutants [166] also merit the assessment of the latter in combination with T&O.

The co-occurrence of T&O and CTX in cyanobacterial blooms raises another important question: do T&O compounds have an impact on the production of CTX? A possibility exists that, once produced by cyanobacteria or other organisms, T&O compounds function as quorum sensors and influence the production of CTX in blooms of cyanobacteria. This would potentially increase the risk of exposure to CTX in an indirect way. This is a factor that should be investigated and should include indications of other organisms associated with cyanobacterial blooms in the studies.

## Figures and Tables

**Table 1:** Cyanotoxins and their cyanobacterial sources—summary of intracellular concentrations in cultured strains and occurrence in the environment.

Cyanotoxins	Potential Cyanobacteria Producer	Cyanotoxins Contents in Cultured Strains (Range)	Occurrence in Water Environments
Microcystins	*Anabaena* sp. *Anabaenopsis* sp. *Annamia toxica* *Aphanocapsa* sp. *Arthrospira* sp. *Calothrix* sp. *Dolichospermum (Anabaena*) sp. *Fischerella* sp. *Haphalosiphon hibernicus* *Leptolyngbya* sp. *Merismopedia* sp. *Microcystis* sp. *Nostoc* sp. *Oscillatoria* sp. *Phormidium* sp. *Planktothrix agardhii* *Planktothrix rubescens* *Plectonema* sp. *Pseudanabaena* sp. *Radiocystis* sp. *Synechococcus* sp.	<0.1–700 mg/g dw <0.3–7 μg/mm^3^ ~2–854 fg/cell	Average in the pelagic water outside scums: <1 up to (not frequently) several tens of μg/L In surface blooms and scums reported maximum values of up 124 mg/L
Nodularin	*Nodularia spumigena* *Nodularia* sp. *Nostoc* sp. *Iningainema pulvinus*	100–700 mg/g dw	In blooms: from 3.5 to 18 mg/g dw In the open water: a few μg up to 18 mg/L in surface blooms
Cylindrospermopsin	*Anabaena lapponica* *Aphanizomenon flosa-quae* *Aphanizomenon gracile * *Chrysosporum* (*Anabaena*) *bergii* *Chrysosporum* (*Aphanizomenon*) *ovalisporum* *Dolichospermum* spp. *Hormoscilla pringsheimii* *Microseira* (*Lyngbya*) *wollei* *R. curvata* *R. mediterranea* *Raphidiopsis* (*Cylindrospermopsis*) *raciborskii* *Umezakia natans*	~1–279 mg/g dw nd—9 μg/mm^3^ 0.3–1.6 fg/cell	<1–10 μg/L, rarely up to 800 μg/L Australia 10–100 up to 800 μg/L Mediterranean regions <10 up to 202 μg/L America and regions in Northern Europe <10 up to 18 μg/L
Anatoxin-a	*Anabaena mendotae* *Blennothrix* *Chrysosporum* (*Aphanizomenon*) *ovalisporum* *Cuspidothrix* *Cylindrospermum*, *D. flosaquae * *D. lemmermannii* *Dolichospermum* (*Anabaena*) *circinale* *Dolichospermum* (*Anabaena*) *flos-aquae* *Kamptonema* *Microcoleus* *Oscillatoria* *Phormidium* *Planktothrix* *Raphidiopsis* (*Cylindrospermopsis*) *Tychonema*	0.003–13 mg/g dw 9.4–400 fg/cell	13–1430 μg/L 0.002–8 mg/g dw USA: nd (most sample) up to 1170 μg/L Europe: range nd-13.1 μg/L max: 444 μg/L Australia: up to 25 μg/L. in bloom: 4.4 mg/g dw Africa: bloom and scum: 1.26 mg/g dw
Homoanatoxin-a	*Dolichospermum/Anabaena* *Kamptonema* (*Oscillatoria*) *formosum* *Microcoleus* (*Phormidium*) *autumnalis * *Oscillatoria * *Raphidiopsis mediterranea*	437 fg/cell; ATXeq Frequently nd	0.44 μg/g ww 34–2118 μg/L
Guanitoxin	*Dolichospermum* (*Anabaena*) *flos-aquae * *D. lemmermannii * *D. spiroides*	nd—0.74 mg/g dw	3.3 mg/g dw
Saxitoxins	*Aphanizomenon* *Dolichospermum* (*Anabaena*) *Microseira (Lyngbya) wollei* *Planktothrix* *Raphidiopsis* (*Cylindrospermopsis*) *raciborskii* *Scytonema* *Sphaerospermopsis torques-reginae*	0.010–2553 μg/g dw 0.77–34.6 fg/cell	3.14–1000 μg/L 0.0005–4.47 mg/g dw 0.07–0.17 fg/cell

Summary of data from [5]. Former genera names are in parentheses. The reported ranges are not related to single producing species but to the values of specific CTX as reported in the literature. For detailed data, see the different chapters of [5,16,26]. Dw—dry weight; ww—wet weight; nd—not detected; ATXeq—anatoxin equivalent.

**Table 2:** Health-Based Guidance Values (HBGV) and other recommended values for various exposure scenarios (µg/L).

Cyanotoxins	Drinking Water (for Chronic Lifetime Exposure)	Drinking Water (Short-Term Exposure ≈2 Weeks) ^3^	Recreational Water (Short-Term Exposure)
Microcystins ^1^	1	12	24
Cylindrospermopsin ^1^	0.7	3	6
Anatoxin-a ^2^	Insufficient information to develop a long-term health-based GV	30	60
Guanitoxin	No toxicological data available (New Zealand has established a limit as provisional maximum acceptable value of 1 μg/L)		
Saxitoxin	Insufficient information to develop a long-term health-based GV	3 ^4^	30

^1^ Provisional health-based GV; ^2^ Although a GV cannot be derived due to inadequate data, a “bounding value”, or provisional health-based reference value, can be derived for short-term exposure to guide actions and responses by water suppliers and health authorities; ^3^ short-term GV refers to the limit of exposure for about 2 weeks, a suitable period of time until measures can be implemented to achieve concentrations < lifetime GV [5]; ^4^ acute exposure. All data from [5].

**Table 3:** Taste and odor compound producers as identified by molecular and/or chemical methods (production), T&O occurrence and co-occurrence of cyanotoxins in cyanobacterial cultures and in field studies.

Habitat	Producing Cyanobacteria	T&O Compounds [Co-Production/Co-Occurrence of Cyanotoxins]			Ref.	A
			Molecular Identification	Production		
Cultures						
Pl	*Dolichospermum circinale* straight (LC006113)	GEO	*gys*	0.01–0.09 ng/µg Chl-*a*	[81]	Yes
		[Total MCs]	*mcyA*	[0.1–0.2 ngMC/µg Chl-*a*]		
Pl	*Dolichospermum circinale* coiled (LC006112)	GEO	*gys*			
			No *mcyA*			
B	*Oscillatoria limosa* (LC178838)	MIB	*mibC*	0.17 ng/µg Chl-*a*		
			No *mcyA*			
Pl	*Coelosphaerium* sp. G2	GEO		Yes	[76]	Yes
Pl	*Coelosphaerium* sp. G2	GEO	*geoA* +		[77]	
	*Coelosphaerium* sp.S3C5		*geoA* −			
	G2 and S3C5 are morphologically identical					
Pl	*Anabaena* sp. Chusori	GEO; extracellular		1–2 pg/cell	[82]	Yes
	*Anabaena sp.* NIER			7–12 pg/cell		
	*Anabaena* sp. FACHB-1384	MIB				
	*Planktothrix* FACHB-1374			~400 pg/cell		
Pl	*Planktothrix* FACHB-1375	MIB		1.6 pg/cell	[83]	Yes
Pl	*Anabaena* sp. CRKS33	GEO	metagenomic analysis		[84]	Yes
		[Bacteriocins]				
		[Aeruginosin]				
Pl	*Dolichospermum circinale* AWQC131C	GEO				
		[Bacteriocins]				
		[Aeruginosin]				
		[STX]				
Pl	*Dolichospermum circinale* AWQC310F	GEO				
		[Bacteriocins]				
		[Aeruginosin]				
		[Cyanobactin]				
Pl	*Aphanizomenon flos-aquae* NIES-81	GEO				
		[Anabaenopeptin]				
		[Bacteriocins]				
Pl	*Dolichospermum* DEX189	GEO	metagenomic analysis		[85]	Yes
		[Anabaenopeptin]				
	*Cylindrospermum stagnale* PCC 7417 *Oscillatoria* sp. 193 *Oscillatoria* sp. PCC 6506	GEO	*geo* *A*	Yes	[78]	
		[ATX] [dihydroanatoxin-a] [homoanatoxin-a]		Data from literature		
L	*Nostoc* sp. UK18aI *Nostoc* sp. UK222II_C *Nostoc* sp. UKK_S60	GEO	*geo* *A*	Yes		
		[MCs]		Data from literature		
	*Dolichospermum smithii* NIES-824	GEO	*geo* *A*	0.7 ng/µg Chl-*a*	[86]	Yes
S	*Microcoleus asticus* sp. nov.	GEO	*geoA*	Yes	[75]	Yes
S	*Microcoleus vaginatus* (Axenic, from soil)	MIB		10–100 ng/L	[87]	Yes
	*Phormidium retzii* (Axenic, from a river)	GEO		10–140 ng/L		
Pl	*Planktothricoides raciborskii*	MIB		3–52 ng/L	[88]	Yes
B	*Oscillatoria limosa* CHAB 7000	GEO	*geo*	140 ng/µg Chl-*a* ^1^ 180 ng/µg Chl-*a* ^2^	[89]	Yes
B	*Phormidium ambiguum* AWQC-PHO021	GEO		3.79 × 10^4^ ng extracel/L in stationary phase	[73,90]	Yes
		[CYN] [deoCYN]		[739 ng/mg dw] [107 ng/mg dw]		
B	I018-018 Unidentified	GEO		8 ng/g ww	[72]	Yes
	I018-002 *Oscillatoria curviceps*			17 ng/g ww		
	I018-046 *Anagnostidinema amphibium*			43 ng/g ww		
	I018-044 *Anagnostidinema acutissimum*			85 ng/g ww		
	I018-031 *Phormidium papyraceum*			167 ng/g ww		
	I018-030 *Leptolyngbya frigida*			211 ng/g ww		
	I018-042 2 filaments—*Phormidium* and *Geitlerinema*			489 ng/g ww		
	I018-001 *Anagnostidinema acutissimum*			1.141 ng/g ww		
	I018-028 *Nodosilinea bijugata*			4.466 ng/g ww		
	I018-043 *Anagnostidinema acutissimum*			6.397 ng/g ww		
	I018-029 2 filaments—*Leptolyngbya* and *Geitlerinema*			16.559 ng/g ww		
	I018-047 *Phormidium lividum*			8 ng/g ww		
	I018-033 2 filaments—*Phormidium* and *Leptolyngbya*	MIB		4.471 ng/g ww		
	I018-023 *Phormidium lusitanicum*	MIB		223 ng/g ww		
		[ATX]		[0.049 ng/g ww]		
	I018-003 *Potamolinea aruginocaerulea*	GEO		8 ng/g ww		
		[ATX]		[0.95 ng/g ww]		
	I018-006 *Anagnostidinema pseudacutissimum*	MIB		26 ng/g ww		
		[ATX]		[0.27 ng/g ww]		
	I018-034 *Geitlerinema sulphureum*	MIB		75 ng/g ww		
		[ATX]		[0.011 ng/g ww]		
	I018-004 *Scytonema crispum*	[MCs]		[80.44 ng/g ww]		
	I018-015 *Geitlerinema acuminatum*	[ATX]		[0.014 ng/g ww]		
	I018-039 *Geitlerinema sulphureum*	[ATX]		[0.014 ng/g ww]		
Pl, B	*Pseudoanabaena galeata* TWNCKU13 *Pseudanabaena galeata* TWNCKU14	MIB	*mib* *C*	5.96 to 51.1 (fg/cell)	[91]	Yes
Pl	*Anabaena ucrainica* CHAB 1434 Nostocales	GEO	*geo*	Yes	[79]	Yes
Pl	*Anabaena planctonica* SDZ-1 Nostocales					
Pl	*Anabaena circinalis* CHAB 3585 Nostocales					
B	*Anabaena minutissima* FACHB 250 Nostocales					
Pe	*Calothrix* sp. CHAB 2384 Nostocales					
B	*Cylindrospermum* sp. CHAB 2115 Nostocales					
Pe, S	*Nostoc commune* FACHB 261 Nostocales					
Pl	*Nodularia* sp. Su-A Nostocales					
Pl	*Aphanizomenon* sp. CHAB 1684 Nostocales					
Pl	*Aphanizomenon gracile* CHAB 2417 Nostocales					
Pe, S	*Nostoc flagelliforme* CHAB 2816 Nostocales					
Pe	*Scytonema* sp. CHAB 3651 Nostocales					
Pe	*Tychonema bourrellyi* CHAB 663 Oscillatoriales					
Pe, S	*Lyngbya kuetzingii* FACHB 388 Oscillatoriales					
Pe	*Phormidium* sp. D6 Oscillatoriales					
Pe, S	*Leptolyngbya bijugata* A4 Oscillatoriales	GEO & MIB producer		Yes		
Pl	*Microcystis aeruginosa* FACHB-905	β-cyclocitral		up to 277.8 µg/L	[92]	
		[MC-LR (dissolved)]		[up to 1.7 µg/L]		
	*Pseudanabaena sp.* FACHB-1277	MIB		up to 178.9 µg/L		
	*Anabaena*	GEO		up to 10 ng/L	[80]	Yes
		MIB		up to 12 ng/L		
	*Oscillatoria*	GEO		up to 7 ng/L		
		MIB		up to 7 ng/L		
	*Microcystis*	GEO		up to 4 ng/L		
		MIB		up to 4 ng/L		
		[MC-LR]		[2.57–23.71 μg/L]		
**Field studies**						
	*Microcystis aeruginosa*	β-cyclocitral		0–538 ng/L	[93] ^3^	
		β-ionone		0–50.44 ng/L		
		GEO		0–11.29 ng/L		
		[MCs]		[0–35.42 μg/L]		
	*Anabaena spiroides* *Microcystis* sp. (mainly *aeruginosa*)	GEO		7.1 μg/L (0.1 pg/cell average)	[94]	
		[MC-RR]		[1.56 μg/L]		
		[MC-LR]		[0.544 μg/L]		
		[MC-YR]		[0.066 μg/L]		
		[ATX]		[0.184 μg/L]		
	Lake Taihu—no analysis of cyanobacteria	GEO		0–37.9 ng/L	[95] ^4^	
		MIB		0–832.9 ng/L		
		β-cyclocitral		0–1706.9 ng/L		
		β-ionone		0–255.2 ng/L		
		[MCs]		[up to 8.716 μg/L]		
		[CYNs]		[up to 0.623 μg/L]		
		[STXs]		[0–0.338 μg/L]		
	*Microcystis * *Dolichospermum*	β-cyclocitral, β-ionone, MIB, GEO		yearly max 250.7 ng/L	[13]	
		[MCs]		[8.86 µg/L]		
B-Mats	Benthic cyanobacteria	GEO	*geoA*		[73]	
		MIB	*MIB synthase gene*			
		[CYNs]	[*cyrA*]			
		[STXs]	[*stx*]			
		[MCs]	[*mcyE*]			
B-Mats	Benthic cyanobacteria from Temperate, Sub-tropical, Tropical regions	GEO	*geoA*		[72]	
		MIB	*MIB synthase gene*			
		[ATX]	[*anaF*]			
		[STX]	[*stxA*]			
		[MC]	[*mcyE*]			
		[CYN]	[*CyrA*]			
	*Planktothrix rubescens*	β-ionone		up to 27 ng/L	[96] ^5^	
	*Anabaena*	GEO	*geoA*	8.02–84.00 ng/L	[97]	
	*Microcystis*	[MCs]	[*mcyE*]	[up to 10 µg/L]		
	*Oscillatoria limosa* (tentatively)	MIB	*MIB synthase gene*	up to 9.7 ng/L		
	species or genera not reported	GEO		max 110 ng/L	[98] ^6^	
		[MCs]		max 7.3 µg/L		
	*Anabaena* *Aphanizomenon* *Microcystis* *Cylindrospermopsis*	GEO		max 0.86 µg/L	[99] ^7^	
		MIB		max 0.06 µg/L		
		[MCs]		[max 19,000 µg/L]		
		[ATX]		[max 9.5 µg/L]		
	*Microcystis* sp. *Cylindrospermopsis* sp. *Anabaena* sp. *Aphanizomenon* *Pseudanabaena*	GEO, MIB, MCs, CYN, STX			[14] ^8^	
	Cyanobacteria genus not reported	MIB		2–30 ng/L	[100]	
		[MCs]		[30–340 ng/L]		

A: new producers not reported in [22]. Pl = planktonic; B = benthic; Pe = periphytic, living attached to underwater surfaces; L = lichen; S = soil; GEO = geosmin; MIB = 2-methylisoborneol; MC = microcystins; STX = saxitoxins; ATX = anatoxin-a; CYN = cylindrospermopsin; deoCYN = deoxy-cylindrospermopsin; MC-LR = microcystin-LR; *geo*; *geoA*; *gys* = GEO synthase gene; *mcyA*; *mcyE* = microcystin synthetase genes; *mibC* = MIB synthase gene; *cyrA* = cylindrospermopsin synthetase gene; *stx*; *stxA* = saxitoxin synthesis genes; *anaF* = anatoxin synthetase gene; dw = dry weight; ww = wet weight. ^1^ T = 25 °C, highest GEO, highest growth; ^2^ 10 µmol phot/m^2^/s highest GEO, lowest growth. ^3^ Correlation between β-cyclocitral, β-ionone from field samples with intracellular and extracellular MCs. ^4^ Positive correlations between MC and β-cyclocitral and β-ionone; positive correlation between CYN and MIB. ^5^ Correlation in the field in Lake Zurich between *P. rubescens* and β-ionone. ^6^ sampling from 2001–2016 in USA; co-occurrence GEO and MICs only 20% of sampling; no correlation. ^7^ 23 lakes USA. Co-occurrence of MCs and GEO 87%; MCs and MIB 39%; ATX and GEO 100%; ATX and MIB 43%. ^8^ 21 drinking water treatment plant. 80% of the analyzed samples (n = 25) saw the co-occurence of at least one cyanotoxin (mainly CYN, but also MCs) with GEO or MIB..
